# Supplementary material for: The Common and Unique Pattern of Microbiome Profiles among Saliva, Tissue, and Stool Samples in Patients with Crohn’s Disease
Source: Microorganisms. 2022 Jul 20;10(7):1467. doi: 10.3390/microorganisms10071467 (PMC9320459; doi:10.3390/microorganisms10071467)
Supplement: Supplementary file 1 [file microorganisms-10-01467-s001.zip › microorganisms-1800595-supplementary.pdf]

# Supplementary Figure

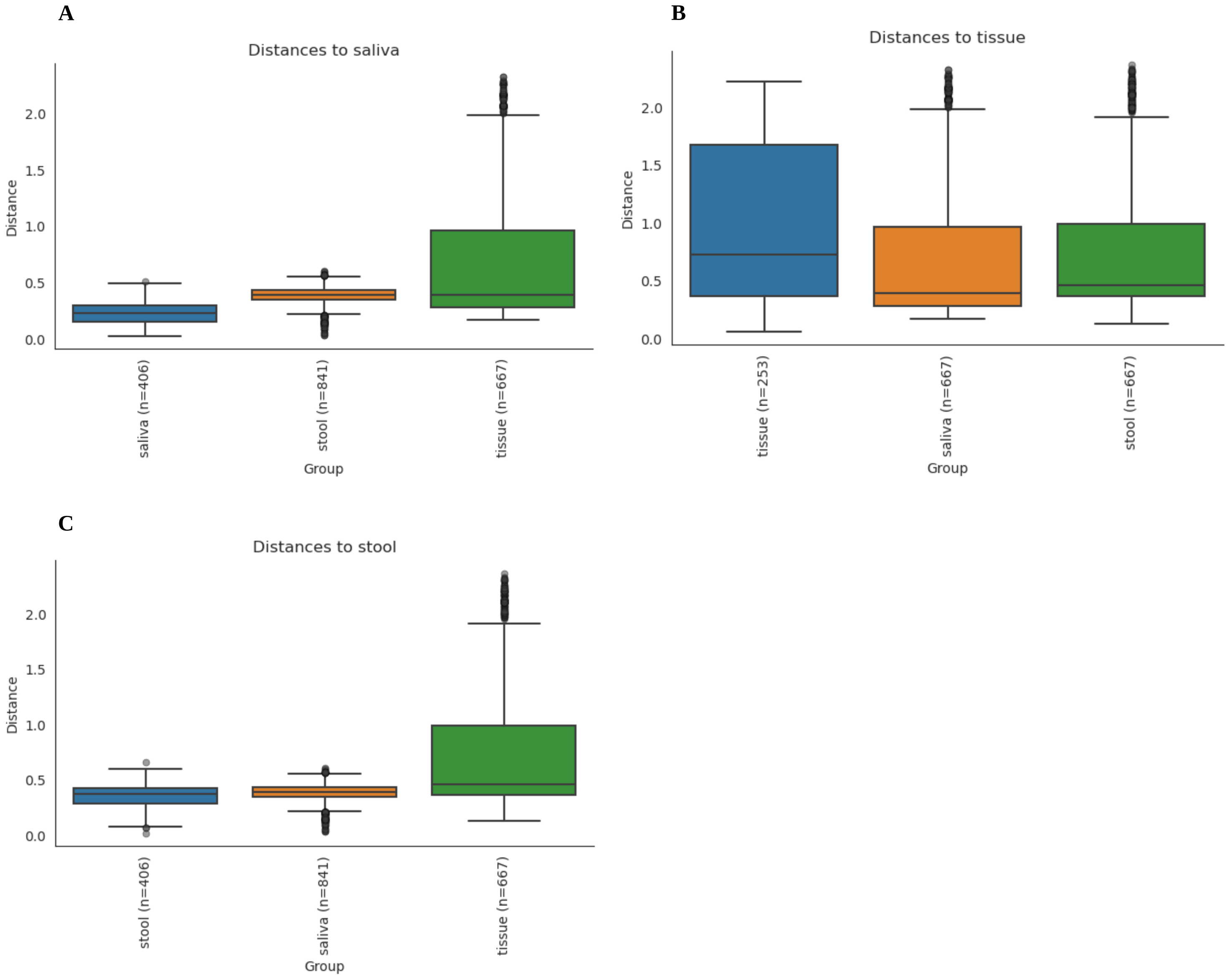


**Supplementary Figure S1.** Beta diversity, weighted UniFrac distance to saliva **(A)**, tissue **(B)**, and stool **(C)** of microbiome in saliva, tissue, and stool of CD patients. Overall distribution patterns are similar to unweighted version, while distances within tissue sample group are maxed and varied distances among members within tissue sample group (median around 0.73, max over 2.2, dominant against other two; saliva with median around 0.40, and stool with median around 0.47) can be explained with existence of subgroup found in UMAP (Fig 3A) based on microbial abundances of each sample, located down left separately, increasing in-group distances of tissue sample group.
